# Multi-Objective Optimization Design and Test of Compound Diatomite and Basalt Fiber Asphalt Mixture

**DOI:** 10.3390/ma12091461

**Published:** 2019-05-06

**Authors:** Yongchun Cheng, Liding Li, Peilei Zhou, Yuwei Zhang, Hanbing Liu

**Affiliations:** College of Transportation, Jilin University, Changchun 130025, China; chengyc@jlu.edu.cn (Y.C.); lild17@mails.jlu.edu.cn (L.L.); ywzhang@jlu.edu.cn (Y.Z.); lhb@jlu.edu.cn (H.L.)

**Keywords:** asphalt mixture, orthogonal experimental design, grey correlation grade analysis, diatomite, basalt fiber, pavement performance, scanning electron microscope

## Abstract

This study focuses on improving the performance of asphalt mixture at low- and high- temperature and analyzing the effect of diatomite and basalt fiber on the performance of the asphalt mixture. Based on the L_16_(4^5^) orthogonal experimental design (OED), the content of diatomite (D) and basalt fiber (B) and the asphalt-aggregate (A) ratio were selected as contributing factors, and each contributing factor corresponded to four levels. Bulk volume density (γ_f_), volume of air voids (VV), voids filled with asphalt (VFA), Marshall stability (MS) and splitting strength at −10 °C (S_b_) were taken as the evaluation indexes. According to the results of the orthogonal experiment, the range analysis and variance analysis were used to study the effect of the diatomite content, basalt fiber content and asphalt-aggregate ratio on the performance of the asphalt mixture, and the grey correlation grade analysis (GCGA) was used to obtain the optimal mixing scheme. Furthermore, the performance tests were conducted to evaluate the performance improvement of asphalt mixtures with diatomite and basalt fibers, and the scanning electron microscopy (SEM) tests were carried out to analyze the mechanism of diatomite and basalt fibers in asphalt mixtures. The results revealed that the addition of diatomite and basalt fiber can significantly increase the VV of asphalt mixture, and reduce γ_f_ and VFA; the optimal performance of the asphalt mixture at high- and low-temperature are achieved with 14% diatomite, 0.32% basalt fibers and 5.45% asphalt-aggregate ratio. Moreover, the porous structure of diatomite and the overlapping network of basalt fibers are the main reasons for improving the performance of asphalt mixture.

## 1. Introduction

With the increase of the traffic in recent years, the pavement performance of the matrix asphalt mixture has been difficult to meet the requirement for long-term service of pavements. Therefore, a large number of asphalt modifiers are used to improve the pavement performance of the asphalt mixture. Ait-Kadi et al. [1,2,3] studied the mechanical performance of polymer modified asphalt using the dynamic mechanical analysis, softening point and Fraass breaking point. The result showed that the overall properties of asphalt could be enhanced by adding the polymer. Khabaz et al. [4] adopted the molecular dynamics simulations to analyze the volumetric, structural and dynamic properties of styrene−butadiene rubber modified asphalt. The results implied that polymeric additives could enhance the dynamic mechanical performance of asphalt without affecting its volumetric performance. Zhu et al. [5] reported that the polymer modifiers had the shortcomings of high cost, low aging resistance and difficult storage. Although the polymer modifiers can enhance the performance of asphalt and asphalt mixtures to some extent, they still have some drawbacks. Diatomite and basalt fiber are often used as two eco-friendly modifiers to enhance the performance of asphalt mixtures [6,7,8,9]. The addition of diatomite and basalt fiber can not only significantly enhance the performance of asphalt mixtures, but also have simpler construction conditions and technologies than some polymer modifiers [7,10,11]. Hence, researchers pay more attention to them.

Tan [6] evaluated the effect of the diatomite content on the low-temperature performance of the asphalt mixture. The low-temperature performance of the diatomite modified asphalt mixture was better than that of the base asphalt mixture. Yang [7] investigated the effect of the diatomite content on the performance of the asphalt mixture. The result showed that diatomite could significantly enhance the high-temperature performance of asphalt mixtures, but had little effect on the low-temperature performance of asphalt mixtures. Zhang [12] investigated the effect of diatomite content on the low-temperature performance of the asphalt mixture. The addition of diatomite resulted in the hardening of the asphalt and decreased its ductility. Wu et al. [13,14,15] studied low-temperature and fatigue performance of the basalt fiber modified asphalt mixture. The results implied that the basalt fiber significantly enhanced the low-temperature performance and fatigue resistance of the asphalt mixture. Fan and Celauro [11,16] studied the influence of basalt fiber on the high-temperature performance of the asphalt mixture. The results revealed that the rutting and stability of the asphalt mixture were improved by adding diatomite.

It can be found from the above that diatomite and basalt fiber can effectively enhance the performance of the asphalt mixture. To further reinforce the performance of the asphalt mixture, Cheng and Davar et al. have made some previous studies on the performance of the asphalt mastic and asphalt mixture modified with diatomite and basalt fibers [11,17,18,19]. Cheng et al. [10,19] pointed out that the diatomite and basalt fiber compound modified asphalt mastic can compensate for the shortcomings of the high-temperature performance of the basalt fiber modified asphalt mastic, and the diatomite and basalt fiber can strengthen the performance of the asphalt mixture. Davar et al. [17] reported that the addition of basalt fiber could significantly reinforce the fatigue life of the diatomite modified asphalt mixture, and the simultaneous use of diatomite and basalt fiber can compensate for shortcomings of the asphalt mixture at low-temperature. Jia [18] noted that the compound addition of diatomite and basalt fibers could improve the resistance to rutting and water damage of recycled hot mix asphalt mixtures.

It can be seen from the previous studies that the combination of diatomite and basalt fiber can significantly enhance the performance of the asphalt mixture. However, the optimum content of diatomite and basalt fiber in the asphalt mixture was directly given by experience. Little work has been done on determining the optimum addition of diatomite and basalt fibers in asphalt mixtures. Moreover, due to the different characteristics of diatomite and basalt fibers on the performance of asphalt mixtures, it is necessary to consider not only their volumetric properties, but also the high- and low-temperature performance in the study of the optimum content of diatomite and basalt fiber in the asphalt mixture. 

The orthogonal experimental design (OED) as a scientific method can be used to study the experimental design of the diatomite and basalt fiber compound modified asphalt mixture (DBFAM) in multi-factor and multi-level arrangements [20,21]. Moreover, since the optimum content of diatomite and basalt fiber is determined by the compound index relating to multiple properties, it requires some mathematical methods to be applied to analyze the multi-objective orthogonal experimental results. The grey correlation grade analysis (GCGA) is a suitable mathematical method that can be used to solve such problems with incomplete information, multi-input and discrete data [22,23,24,25].

In this paper, the multi-objective OED and the GCGA were used to quantitatively study the optimum addition of diatomite and basalt fiber and the optimal asphalt-aggregate ratio. The asphalt mixture performance tests were carried out to check the improvement of the asphalt mixture performance by diatomite and basalt fiber. The scanning electron microscopy (SEM) tests were also performed to analyze the improvement mechanism of the performance of diatomite and basalt fiber on the asphalt mixture. This work could provide some references for the practical application of diatomite and basalt fiber in pavement engineering.

## 2. Materials and Methods 

### 2.1. Materials

The asphalt, A-90#, supplied by Pan Petrochemical Industry was used in this paper, and its properties are shown in Table 1. The diatomite originating from Changbai Mountain in the Jilin province was sieved by 0.075 mm sieve, and the undersize was used for subsequent experiments. The chemical composition of diatomite is presented in Table 2. The basalt fibers were supplied from the Jiuxin Basalt Industry Co, Ltd, Jilin province, with a diameter of 10–13 μm and a length of 6 mm, and its properties are presented in Table 3. The aggregate used in the test was produced form basalt by the Yingshan Mountain, Liaoyuan City, Jilin Province. The filler aggregate was produced in Shuangyang City, Jilin province. With the nominal maximum aggregate size of 13 mm shown in Table 4 and the different mix proportion, 16 groups of asphalt mixtures were fabricated according to the Standard Test Methods of Bitumen and Bituminous Mixtures for Highway Engineering (JTG E20-2011) [26].

### 2.2. Experimental Scheme

In this paper, the OED with three factors and four levels L_16_(4^3^) was designed by choosing the diatomite content (D) (added in substitution to mineral filler), basalt fiber content (B) (relative to mineral mass ratio) and asphalt-aggregate ratio (A) as orthogonal factors [27,28,29]. Their levels are determined as presented in Table 5. Bulk volume density (γ_f_), volume of air voids (VV), voids filled with asphalt (VFA), Marshall stability (MS) and splitting strength at –10 °C (S_b_) were used to determine the optimum content of diatomite and basalt fibers in the asphalt mixture and the optimum asphalt-aggregate ratio of the asphalt mixture.

### 2.3. Experimental Methods

According to JTG E20-2011, the performance of the DBFAM and its base asphalt mixture (AM) was tested [26].

#### 2.3.1. Rutting Tests and the Uniaxial Compression Creep Test

The rutting tests at 60 °C and the uniaxial compression creep tests at 50 °C were carried out to evaluate the high-temperature resistance to permanent deformation of the asphalt mixture. For the rutting tests, a contact pressure of 0.7 MPa was applied on the square asphalt mixture slab with a size of 300 mm × 300 mm × 50 mm after 5 h under 60 °C ± 0.5 °C dry condition. The speed of the wheel with a pressure of 0.7 MPa is 42 ± 1 times/min along the center line of the slab. The loading and rolling are continued for 60 min at 60 °C. For the uniaxial compression creep tests, a contact pressure of 10% uniaxial compressive strength was applied on the cylindrical asphalt mixture specimen (100 mm in diameter and 100 mm in height) after 5-h drying at 50 °C [30]. The loading is continued for 1 h at 50 °C.

The dynamic stability (DS) of the rutting test is calculated according to Equation (1).

(1) $$DS=\frac{(t_{2}-t_{1})\times N}{d_{2}-d_{1}}=\frac{15\times 42}{d_{2}-d_{1}}$$

where *N* is the wheel moving speed, 42 times/min; *d_1_* is the tracking depth at *t_1_* (45 min), mm; *d_2_* is the tracking depth at *t_2_* (60 min), mm.

The creep rate ($\varepsilon_{speed}$) of the uniaxial compression creep tests was computed by Equation (2).

(2) $$\varepsilon_{speed}=\frac{\left(\varepsilon_{2}-\varepsilon_{1})/(t_{2}-t_{1}\right)}{\sigma_{0}}$$

where *t_1_* and *t_2_* is are the start time and end time of the creep stabilization phase, respectively; *ε_1_* and *ε_2_* are strains corresponding to *t_1_* and *t_2_*, respectively; *σ_0_* is the creep stress.

#### 2.3.2. Splitting Tests and Three-Point Bending Test

The splitting tests at −10 °C and three-point bending tests at −10 °C were conducted to evaluate the resistance to the cracking ability of the asphalt mixture at low-temperature. Before the splitting tests and three-point bending tests, the standard Marshall specimens (101.6 mm in diameter and 63.5 mm in height) and the beam specimens (250 mm × 30 mm × 35 mm) were conditioned at −10 °C for 4 h. The loading speed of splitting tests and three-point bending tests were 1 mm/min and 50 mm/min, respectively. And the tests were performed at −10 °C ± 0.5 °C.

The splitting tensile strength (*R_T_*), the tensile strain (*ε_T_*) and the stiffness modulus (*S_T_*) for the splitting test at −10 °C are calculated by Equation (3)– Equation (5).

(3) $$R_{T}=\frac{0.006287\times P_{T}}{h}$$

(4) $$\varepsilon_{T}=\frac{Y_{T}\times (0.0307+0.0936\times \mu )}{\left(17.94-0.314\times \mu \right)}$$

(5) $$S_{T}=\frac{P_{T}\times (3.588-0.0628\times \mu )}{h\times Y_{T}}$$

where *P_T_* is the maximum test load, N; *h* is the height of Marshall specimens, mm; *Y_T_* is the total vertical deformation corresponding to the maximum breaking load, mm; *μ* is Poisson’s ratio, which is 0.25.

The maximum bending strength (*R_B_*), the maximum bending strain (*ε_B_*) and the bending stiffness modulus (*S_B_*) for the three-point bending test were computed according to Equation (6)–Equation (8).

(6) $$R_{B}=\frac{3\times L\times P_{B}}{2\times b\times h^{2}}$$

(7) $$\varepsilon_{B}=\frac{6\times h\times d}{L^{2}}$$

(8) $$S_{B}=\frac{R_{B}}{\varepsilon_{B}}$$

where *P_B_* is the displacement inducing force, N; *L* is the spanning length, mm; *h* and *b* are the height and width of the beam respectively, mm; *d* is the mid-span deflection, mm.

#### 2.3.3. Immersion Marshall Test and Freeze-Thaw Splitting Test

Immersion Marshall tests and freeze-thaw splitting tests were adopted to evaluate the moisture susceptibility of the asphalt mixture. The standard Marshall specimens of Immersion Marshall tests were prepared at 60 °C for 48 h. Then, the Marshall stability of the conditioned and unconditioned specimens was tested at 60 °C with the loading speed of 50 mm/min. The standard Marshall specimens of freeze-thaw splitting tests were prepared at −18 °C ± 2 °C for 16 h and 60 °C ± 0.5 °C for 24 h. Subsequently, the splitting strength of the conditioned and unconditioned specimens was tested at 25 °C for 2 h with the loading speed of 50 mm/min.

The residual stability (*MS_0_*) for the immersion Marshall test was calculated according to Equation (9).

(9) $$MS_{0}=\frac{MS_{1}}{MS}\times 100$$

where *MS_1_* is the average Marshall stability of conditioned specimens, kN; *MS* is the average Marshall stability of unconditioned specimens, kN.

The tensile strength ratio (*TSR*) for the freeze-thaw splitting test was computed by Equation (10).

(10) $$TSR=\frac{R_{T2}}{R_{T1}}\times 100$$

where *R_T1_* and *R_T2_* are the average tensile strength of unconditioned specimens and conditioned specimens respectively, MPa. 

#### 2.3.4. SEM Test

The SEM tests (JSM-6460LV, JEOL, Tokyo, Japan) were carried out to observe the microstructure of diatomite and basalt fibers, and the interface of diatomite and basalt fiber with asphalt. The magnification is 500 times to 3000 times the original size.

### 2.4. GCGA Algorithm

To determine the optimum content of diatomite and basalt fibers in the asphalt mixture under multi-objective, the GCGA algorithm was applied to analyze the results of the OED [22,23,24,25]. A grey correlation coefficient between the reference sequence *x_0_* = (*x_0_*(1), *x_0_*(2), …, *x_0_*(5)) and comparative sequences *x_i_* = (*x_i_*(1), *x_i_*(2), …, *x_i_*(5)), $\xi_{i}(k)$, is defined as

(11) $$\xi_{i}\left(k\right)=\frac{\underset{i}{\min}\;\underset{k}{\min}\left|x_{0}\left(k\right)-x_{i}\left(k\right)\right|+0.5\;\underset{i}{\max}\;\underset{k}{\max}\left|x_{0}\left(k\right)-x_{i}\left(k\right)\right|}{\left|x_{0}\left(k\right)-x_{i}\left(k\right)\right|+0.5\;\underset{i}{\max}\;\underset{k}{\max}\left|x_{0}\left(k\right)-x_{i}\left(k\right)\right|}$$

where $\xi_{i}(k)$ represents the correlation coefficient between the *k*-th evaluation index of the *i*-th comparative group and the corresponding reference group. $\underset{i}{\max}\;\underset{k}{\max}\left|x_{0}(k)-x_{i}(k)\right|$ are the maximum difference values between the reference sequence and comparative sequences; $\underset{i}{\min}\;\underset{k}{\min}\left|x_{0}(k)-x_{i}(k)\right|$ are the minimum difference values between the reference sequence and comparative sequences.

Then, the grey correlation grade, $\Gamma_{i}$, is obtained from a weighting-sum of grey correlation coefficient as described the following Equation (12).

(12) $$\Gamma_{i}=A_{r}\xi_{i}(k)+A_{v}\xi_{i}(k)+A_{F}\xi_{i}(k)+A_{M}\xi_{i}(k)+A_{S}\xi_{i}(k)$$

(13) $$A_{r}+A_{v}+A_{F}+A_{M}+A_{S}=1$$

where $A_{r}$, $A_{v}$, $A_{F}$, $A_{M}$ and $A_{S}$ represent the weighting of γ_f_, VV, VFA, MS and S_b_.

## 3. Results and Discussion

### 3.1. Orthogonal Experimental Results

According to the multi-objective OED, 16 groups of asphalt mixtures were fabricated, and γ_f_, VV, VFA, MS and S_b_ were tested and calculated. The test results were shown in Table 6.

### 3.2. Range Analysis

The range analysis method was used to study the degree of influence of the test factors on the test results, and the larger range of test results mean the greater the influence of the test results. The parameter *k_ij_* and range (*R_j_*) were used for the evaluation. The parameter *k_ij_* is defined as the mean value of the evaluation indexes of all levels (*j*, *j* = 1, 2, 3, 4) in each factor (*i*, *i* = D, B, A) [28]. For this L_16_(4^3^) orthogonal experiment, the calculations are as follows (factor D, for example):

(14) $$k_{D1}=(E_{1}+E_{2}+E_{3}+E_{4})/4$$

(15) $$k_{D2}=(E_{5}+E_{6}+E_{7}+E_{8})/4$$

(16) $$k_{D3}=(E_{9}+E_{10}+E_{11}+E_{12})/4$$

(17) $$k_{D4}=(E_{13}+E_{14}+E_{15}+E_{16})/4$$

(18) $$R_{D}=\max(k_{D1},k_{D2},k_{D3},k_{D4})-\min(k_{D1},k_{D2},k_{D3},k_{D4})$$

where *E* is the evaluation indexes, such as the γ_f_, VV, VFA, MS and S_b_.

**Figure 1:**
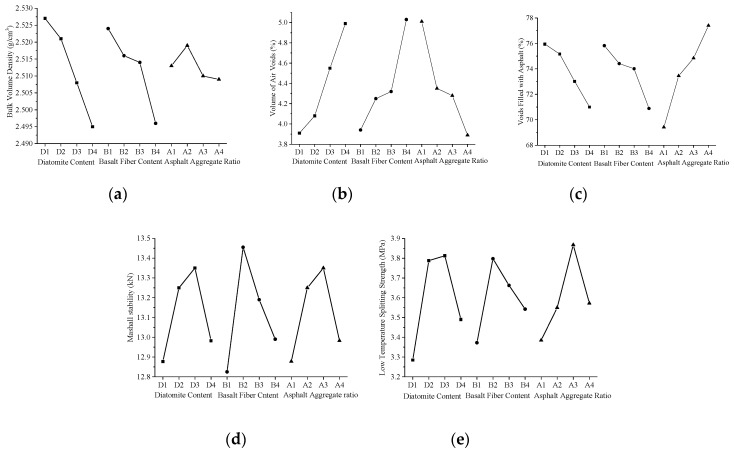
Relationship between mean value of each factor under different evaluation indexes: (**a**) γ_f_; (**b**) VV; (**c**) VFA; (**d**) MS; (**e**) S_b_.

Figure 1 and Table 7 showed the calculation results of k_ij_ and R_j_ of γ_f_, VV, VFA, MS and S_b_. It can be seen from Table 7 that the influence degree of diatomite, basalt fibers, and asphalt-aggregate ratio on different evaluation indexes is quite different. As can be seen from Figure 1a–c, with the increasing of diatomite content and basalt fibers content, γ_f_ and VFA of the asphalt mixture decrease, and VV increases. This implies that the addition of diatomite and basalt fiber could have an adverse effect on γ_f_ and VV of the asphalt mixture, and the filling of asphalt to voids of the mineral aggregate.

With the increasing of the asphalt-aggregate ratio, γ_f_ of the asphalt mixture first increases and then decreases, and reaches its peak at 5.2% asphalt-aggregate ratio (Figure 1a). The voids of the asphalt mixture are increasingly filled with the increase of asphalt, so that VV decreased continuously (Figure 1b), and VFA increased consistently (Figure 1c).

Figure 1d,e show the trend of MS and S_b_ with the level of each factor. It can be seen from the figures that both the MS and S_b_ increase first and then decrease with the increase of the addition of diatomite and basalt fibers. Which means that the reasonable addition content of diatomite and basalt fiber can significantly enhance the high-temperature stability and low-temperature splitting strength.

### 3.3. Variance Analysis

Table 8 shows that variance analysis results of the effect of diatomite, basalt fiber and asphalt-aggregate ratio on γ_f_, VV, VFA, MS and S_b_ of the asphalt mixture. F-value and p-value obtained from the variance analysis can reflect the significance of the influence of each test factors on the evaluation indexes. When F > F_0.05_(3,6) (F_0.05_(3,6) = 4.76) and p-value <0.05, it can be considered that the test factors have a significant impact on the evaluation indexes. It can be seen from Table 8 that diatomite, basalt fibers, and asphalt-aggregate ratio can have significant effects on the five test indicators, but the influence degree of various factors on different test indicators is quite different. Effect on γ_f_ of the asphalt mixture: Diatomite > basalt fibers > asphalt-aggregate ratio; the three factors have similar effects on VV; effect on VFA: asphalt-aggregate ration > diatomite > basalt fibers; effect on MS: Asphalt-aggregate ratio > basalt fibers > diatomite; effect on S_b_: Diatomite > asphalt > basalt fiber.

### 3.4. GCGA

The experiments with three factors and four levels actually have 64 test groups, but only 16 groups of experiments are needed in the OED. The remaining 48 groups are unknown, which constitutes a gray system containing both non-deterministic information and certain information. Moreover, it is difficult for the general algorithm to obtain a reasonable optimal parameter combination for multi-objective requirements. However, the GCGA is a scientific method to evaluate the correlation between factors based on the similarity between the development trends of factors, which can solve the problem of multi-objective optimization design in this research scheme very well [31,32].

Due to the different physical meanings of the evaluation indicators, the dimension of the data is not uniform, and it is not easy to compare, and it is difficult to get the correct conclusion when comparing. The dimensionless processing of the multi-objective test results is needed before GCGA [24,33,34].

In general, to achieve the best performance of the asphalt mixture, volume indexes are required to be close to the target value, and the mechanical indexes are required to reach the maximum. The normalization algorithm of γ_f_, MS and S_b_ are shown in Equation (19). The larger the values of these indicators, the better the performance of the asphalt mixture. The normalization algorithm of VV and VFA are shown in Equation (20). These indicators are assigned an optimal value. The dimensionless results of all indicators are shown in Table 6 (10th to 14th columns in Table 6).

(19) $$x_{i}=\frac{f_{i}-\min\left(f_{1},f_{2},\cdots ,f_{16}\right)}{\max(f_{1},f_{2},\cdots ,f_{16})-\min(f_{1},f_{2},\cdots ,f_{16})}$$

(20) $$x_{i}=\frac{-\left|f_{i}-f_{target}\right|+\max{\left|f_{j}-f_{target}\right|}_{j=1,2,\cdots ,16}}{\max{\left|f_{j}-f_{target}\right|}_{j=1,2,\cdots ,16}-\min{\left|f_{j}-f_{target}\right|}_{j=1,2,\cdots ,16}}$$

where *i* = 1, 2, …, 16. *x_i_* is the dimensionless result of the experimental data of the i-th group; *f_i_* and *f_j_* are the test results of group *i* and *j*, respectively. *f_target_* is the target indicator given in advance, and VV are set to 4% and VFA is set to 70%.

The GCGA first determines the reference sequence and the comparison sequence. In this paper, the maximum values of γ_f_, MS, S_b_, and the target values of VV, VFA in 16 groups of test data are set as reference series, that is, the maximum value of five test indicators after dimensionless in the overall scheme, which is recorded as *x*_0_ = (*x*_0_(1), *x*_0_(2), …, *x*_0_(5)). The dimensionless results of 16 schemes are set as comparison sequences.

According to Equation (11), the grey correlation coefficient $\xi_{i}(k)$ are calculated for the dimensionless test results, and the results are shown in Table 9. In the design of the asphalt mixture proportion, the volume indexes and performance indexes of the asphalt mixture are of equal importance. At present, the design method of the asphalt mixture generally adopts the volume design method, however, the reasonable volume parameter is necessary but is not a sufficient condition for the excellent performance of the asphalt mixture. Therefore, according to the volume design method, this paper adds performance indicators. And the volume indexes and the mechanical indexes are given the same weight to obtain a reasonable optimum content of diatomite and basalt fibers in the asphalt mixture and optimum asphalt-aggregate ratio of the asphalt mixture. The weight of volume indexes are set to $A_{r}$ = 0.1, $A_{v}$ = 0.2, $A_{F}$ = 0.2, and the weight of mechanical indexes are set to $A_{M}$ = 0.25, $A_{S}$ = 0.25. According to the above weight and Equation (12), the grey correlation grade Γ between the reference sequence and comparative sequences are calculated as shown in Table 9. The average correlation grade corresponding to each evaluation and each level is calculated as in Table 10.

**Figure 2:**
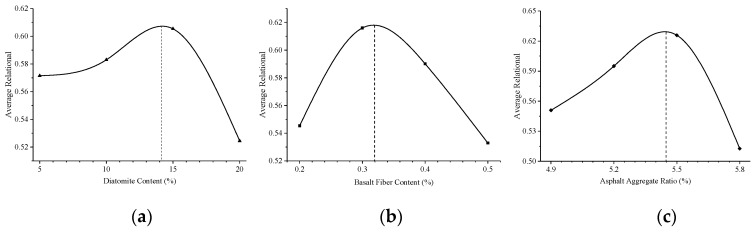
The trend of average correlation grade with the level of each evaluation index: (**a**) The trend of average correlation grade with diatomite content; (**b**) the trend of average correlation grade with basalt fiber content; (**c**) the trend of average correlation grade with asphalt-aggregate ratio content.

The trend of the average correlation grade with the level of each evaluation index is shown in Figure 2. According to the GCGA algorithm, a greater correlation grade means that the corresponding factor level is closer to the optimal value [23]. It can be seen from Figure 2 that the optimal proportion corresponding to each factor is D = 14%, B = 0.32%, and A = 5.45%. Therefore, according to the multi-objective OED, when the diatomite content is 14%, the basalt fiber content is 0.32%, and the asphalt-aggregate ratio is 5.45%, the performances of DBFAM at high- and low-temperature are optimal.

### 3.5. Performance Verification

The optimum proportion of the diatomite, basalt fiber, and asphalt-aggregate ratio is obtained by the GCGA algorithm in the above. Under the optimum proportion, DBFAM and AM were prepared. And the resistance to permanent deformation at high-temperature, anti-cracking performance at low-temperature and moisture susceptibility resistance were tested.

#### 3.5.1. Resistance to Permanent Deformation at High-Temperature

The rutting tests at 60 °C and the uniaxial compression creep tests at 50 °C are used to characterize the high-temperature resistance to permanent deformation of the asphalt mixture modified by diatomite and basalt fiber.

Table 11 shows DS and $\varepsilon_{speed}$ of DBFAM and AM. It can be seen from Table 11 that compared with AM, the DS of DBFAM increases by 853.5 times/min, the anti-rutting deformation ability increases by 54.38%, the $\varepsilon_{speed}$ decreases by 1.242 × 10^−7^ (s·MPa)^−1^, and the permanent deformation resistance at 50 °C increases by 19.67% after adding diatomite and basalt fiber.

It implies that the addition of diatomite and basalt fiber can significantly improve the high-temperature performance of the asphalt mixture, which may be attributed to the fact that diatomite with reasonable content can absorb excess free asphalt in the asphalt mixture, thereby increasing the relative content of structural asphalt, so as to improve the high-temperature resistance to permanent deformation of the asphalt mixture.

#### 3.5.2. Anti-Crack at Low Temperature

The crack-resistance at low-temperature is used to characterize the ability of the asphalt mixture to resist temperature shrinkage cracking in the event of sudden temperature changes or in cold regions. The splitting tests at −10 °C and the three-point bending tests at −10 °C are used to evaluate the effect of diatomite and basalt fiber on the performance of crack-resistance at low-temperature.

Table 12 shows the calculation results of tensile strength, maximum strain and stiffness modulus of DBFAM and AM. It can be found from the splitting test results in Table 12 that the addition of diatomite and basalt fiber increases the tensile strength of the asphalt mixture by 15.72%, the tensile strain of the fracture by 22.44%, and the modulus of failure by 5.38% at low temperature. It can be seen from the results of three-point bending tests in Table 12 that compared with AM, the maximum bending strength of the compound modified asphalt mixture increases by 14.58%, the maximum bending strain increases by 17.40%, and the bending stiffness modulus decrease by 2.54%. When the maximum tensile strain and bending strain are taken as the main evaluation indexes of low-temperature performance, it can be seen that the low-temperature performance of asphalt mixture can be significantly improved by adding diatomite and basalt fiber. This may be attributed to the fact that basalt fibers in asphalt mixtures can play a better role in strengthening, toughening and crack resistance.

#### 3.5.3. Moisture Susceptibility

In the long-term water environment, water molecules continuously invade the interface between the asphalt and aggregate, which will cause asphalt to peel off from the aggregate and seriously damage the mechanical performance of the asphalt mixture. The immersion Marshall tests and the freeze-thaw splitting tests are used to evaluate moisture susceptibility of DBFAM in this paper.

Table 13 shows the calculation results of the two kinds of tests. It can be seen from Table 13 that compared with AM, the immersion residual stability of DBFAM decreases by 0.67%, and the freeze-thaw splitting strength ratio increases by 1.34%.

### 3.6. Analysis of Improvement Mechanism

From the pavement performance test results, it can be seen that the addition of diatomite and basalt fiber can significantly enhance high- and low- temperature performance of the asphalt mixture. To further study the mechanism of diatomite and basalt fibers in asphalt mixtures, the SEM tests of diatomite, basalt fibers, diatomite asphalt and basalt fibers in the asphalt were carried out.

**Figure 3:**
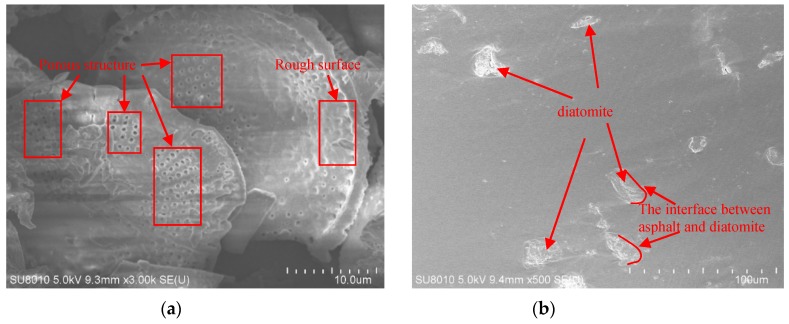

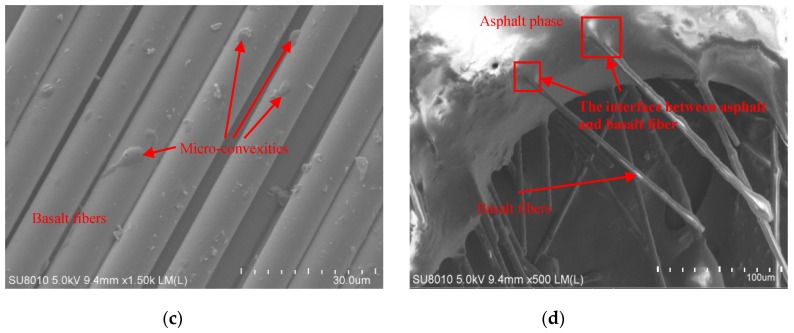
SEM micrographs: (**a**) Diatomite (D); (**b**) diatomite in asphalt; (**c**) basalt fiber (B); (**d**) basalt fibers in asphalt.

The results of SEM are shown in Figure 3. It can be seen from Figure 3a that diatomite particles have a large number of pore structures and their surfaces are uneven. In Figure 3b, it can be found that diatomite can be well dispersed in asphalt and has good wettability with asphalt. Which means that diatomite can adsorb a large amount of free asphalt in the asphalt mixture and increase the relative content of structural asphalt in the asphalt mixture, thereby significantly improving the high-temperature performance of the asphalt mixture. Furthermore, it can be found in Figure 3c that basalt fiber is tiny and has many bumps on the surface. And Figure 3d shows that basalt fibers can be spatially reticulated in the asphalt and well infiltrated by the asphalt. It means that basalt fiber can play a reinforcing and toughening role in asphalt mixtures, which may be the reason why basalt fiber can increase the low-temperature crack resistance of asphalt mixtures.

## 4. Conclusions

In this paper, the multi-objective OED and the GCGA algorithm were applied to determine the optimum proportion of diatomite and basalt fiber in the asphalt mixture. The pavement performance tests were carried out to check the performance of DBFAM, and the SEM tests were used to analyze the improvement mechanism of diatomite and basalt fiber on the performance of the asphalt mixture. Based on the above research, the following conclusions can be drawn.

(1) The multi-objective OED and the GCGA algorithm can be well applied to quantitatively study the optimum proportion of diatomite and basalt fiber in the asphalt mixture, as they can simultaneously consider both high- and low- temperature performance and volume indexes.

(2) Adding diatomite and basalt fiber can increase the VV of the asphalt mixture, and decrease γ_f_ and VFA of the asphalt. Using 14% diatomite, 0.32% basalt fiber and 5.45% asphalt-aggregate ratio can prepare the DBFAM with an optimal performance at high- and low-temperature.

(3) Compared with AM, the anti-rutting and anti-creep of DBFAM increases by 54.38% and 19.67%, respectively; the tensile strain and the maximum bending strain increases by 22.44% and 17.40%, respectively; the residual stability decreases by 0.67%, and the freeze-thaw splitting strength ratio increases by 1.34%.

(4) Diatomite can adsorb a large amount of free asphalt and increase the relative content of structural asphalt in the asphalt mixture, thereby significantly improving the high-temperature performance of the asphalt mixture. Basalt fiber can play a reinforcing and toughening role in asphalt mixtures, which can significantly enhance the low-temperature crack resistance of the asphalt mixture.

## Figures and Tables

**Table 1:** Physical properties of matrix asphalt.

Properties	Value	Standard Value
Density (15 °C, g/cm^3^)	1.016	—
Penetration (25 °C, 0.1 mm)	91.8	80–100
Softening point T_R__&B_ (°C)	46.9	≥45
Ductility (25 °C, cm)	>150	≥100
Viscosity (135 °C, Pa·s)	0.307	—
After TFOT		
Mass loss (%)	0.38	≤±0.8
Residual penetration ratio (25 °C, %)	73.3	≥57
Softening point T_R__&B_ (°C)	49.6	—
Ductility (15 °C, cm)	>120	≥20
Viscosity (135 °C, Pa·s)	0.433	—

**Table 2:** Diatomite chemical composition.

Chemical Composition	SiO_2_	Al_2_O_3_	Fe_2_O_3_	CaO	MgO	TiO_2_	K_2_O
Proportion (%)	85.60	4.50	1.50	0.52	0.45	0.30	0.67

**Table 3:** Properties of basalt fibers.

Properties	Diameter (*μ*m)	Length (mm)	Water Content (%)	Combustible Content (%)	Tensile Strength (MPa)	Tensile Modulus of Elasticity (GPa)	Elongation at Break (%)
Value	10–13	6	0.030	0.56	2320	86.3	2.84

**Table 4:** Aggregate gradation of AC-13.

Sieve size (mm)	0.075	0.15	0.3	0.6	1.18	2.36	4.75	9.5	13.2	16
Percent Passing (%)	6	10	13.5	19	26.5	37	53	76.5	95	100

**Table 5:** Experimental factors and their levels.

Experimental Factors	Symbol	Level 1	Level 2	Level 3	Level 4
Diatomite content	D	5% (D1)	10% (D2)	15% (D3)	20% (D4)
Basalt fiber content	B	0.2% (B1)	0.3% (B2)	0.4% (B3)	0.5% (B4)
Asphalt-aggregate ratio	A	4.9% (A1)	5.2% (A2)	5.5% (A3)	5.8% (A4)

**Table 6:** L_16_(4^3^) orthogonal array and the results.

Groups	Test Factor			Test Result					Non-Dimensional				
	D (%)	B (%)	A (%)	γ_f_ (g/cm³)	VV (%)	VFA (%)	MS (kN)	S_b_ (MPa)	γ_f_	VV	VFA	MS	S_b_
1	5	0.2	4.9	2.541	4.02	74.01	12.46	2.81	1.00	1.00	0.62	0.04	0.00
2	5	0.3	5.2	2.534	3.85	75.85	13.38	3.50	0.89	0.93	0.43	0.61	0.46
3	5	0.4	5.5	2.524	3.80	76.99	13.27	3.58	0.74	0.90	0.31	0.54	0.52
4	5	0.5	5.8	2.508	3.99	76.93	12.4	3.25	0.49	1.000	0.32	0.00	0.30
5	10	0.2	5.5	2.532	3.44	78.76	13.32	3.89	0.86	0.70	0.12	0.57	0.73
6	10	0.3	5.8	2.523	3.36	79.94	13.06	3.91	0.72	0.66	0.00	0.41	0.74
7	10	0.4	4.9	2.520	4.77	70.40	13.27	3.69	0.68	0.59	1.00	0.54	0.59
8	10	0.5	5.2	2.509	4.76	71.55	13.35	3.66	0.51	0.6	0.88	0.59	0.57
9	15	0.2	5.8	2.512	3.73	78.14	12.67	3.57	0.55	0.86	0.19	0.17	0.51
10	15	0.3	5.5	2.506	4.39	74.20	14.02	4.30	0.46	0.80	0.60	1.00	1.00
11	15	0.4	5.2	2.522	4.21	74.06	13.57	3.82	0.71	0.89	0.62	0.72	0.68
12	15	0.5	4.9	2.490	5.86	65.67	13.14	3.56	0.22	0.00	0.59	0.46	0.50
13	20	0.2	5.2	2.511	4.58	72.36	12.85	3.22	0.54	0.70	0.79	0.28	0.28
14	20	0.3	4.9	2.501	5.39	67.63	13.36	3.48	0.39	0.25	0.79	0.59	0.45
15	20	0.4	5.8	2.491	4.49	74.61	12.65	3.56	0.23	0.74	0.56	0.15	0.50
16	20	0.5	5.5	2.476	5.49	69.41	13.07	3.70	0.00	0.20	0.98	0.41	0.60

**Table 7:** The results of range analysis for evaluation indexes.

Evaluation Indexes	γ_f_ (g/cm3)	VV (%)	VFA (%)	MS (kN)	Sb (MPa)
R_D_	0.032	1.074	4.939	0.473	0.528
R_B_	0.028	1.083	4.927	0.630	0.425
R_A_	0.011	1.120	7.976	0.725	0.482

**Table 8:** Variance analysis of the effect of test factors on the test results.

Factor	γ_f_		VV		VFA		MS		S_b_	
	F-Value	P-Value	F-Value	P-Value	F-Value	P-Value	F-Value	P-Value	F-Value	P-Value
D	44.8	1.7 × 10^−4^	34.5	3.5 × 10^−4^	31.7	9.4 × 10^−4^	24.3	3.0 × 10^−5^	81.3	4.5 × 10^−4^
B	31.14	4.7 × 10^−4^	30.8	4.8 × 10^−4^	27.6	3.0 × 10^−4^	36.4	2.1 × 10^−4^	41.5	6.6 × 10^−4^
A	4.9	4.7 × 10^−2^	31.8	4.5 × 10^−4^	71.0	1.2 × 10^−4^	49.8	1.1 × 10^−4^	51.3	4.5 × 10^−5^

**Table 9:** Analysis results of grey correlation degree of objective function.

Number	γ_f_	VV	VFA	MS	S_b_	Γ
1	1.000	0.995	0.569	0.342	0.333	0.582
2	0.823	0.871	0.467	0.559	0.482	0.610
3	0.657	0.832	0.420	0.519	0.509	0.573
4	0.496	1.000	0.422	0.333	0.415	0.521
5	0.783	0.628	0.363	0.536	0.645	0.572
6	0.644	0.594	0.333	0.458	0.656	0.528
7	0.607	0.550	1.000	0.519	0.550	0.638
8	0.504	0.554	0.806	0.547	0.538	0.594
9	0.528	0.781	0.381	0.375	0.505	0.505
10	0.481	0.712	0.556	1.000	1.000	0.802
11	0.631	0.824	0.566	0.643	0.608	0.654
12	0.389	0.333	0.548	0.479	0.502	0.460
13	0.520	0.622	0.709	0.409	0.408	0.522
14	0.448	0.401	0.708	0.551	0.476	0.523
15	0.394	0.659	0.531	0.372	0.502	0.496
16	0.333	0.384	0.962	0.460	0.554	0.556

**Table 10:** Average relevance degree between levels of each factor and object function.

D (%)	Relational	B (%)	Relational	A (%)	Relational
5	0.572	0.2	0.545	4.9	0.551
10	0.583	0.3	0.616	5.2	0.595
15	0.605	0.4	0.590	5.5	0.626
20	0.524	0.5	0.533	5.8	0.513

**Table 11:** Test results of high-temperature permanent deformation resistance.

Tests	Index	AM	DBFAM
Rutting tests	DS (times/min)	1569.6	2423.1
Creep tests under uniaxial compression	$\varepsilon_{speed}$ ((s·MPa)^−1^)	6.313 × 10^−7^	5.071 × 10^−7^

**Table 12:** Test results of cracking resistance at low temperature.

Tests	Mixture	Tensile Strength (MPa)	Destructive Strain (με)	Stiffness Modulus (MPa)
Splitting (−10 °C)	AM	3.69	2923	2173
	DBFAM	4.27	3579	2056
Three-point bending (−10 °C)	AM	8.60	2079	4137
	DBFAM	9.85	2441	4032

**Table 13:** Test results of water stability.

Mixture	Immersed Marshall Test			Freeze-Thaw Splitting Test		
	MS (kN)	MS_1_ (kN)	MS_0_ (%)	R_T1_ (MPa)	R_T2_ (MPa)	TSR (%)
AM	13.19	12.62	95.68	1.258	1.153	91.65
DBFAM	13.83	13.14	95.01	1.155	1.074	92.99
